# Impact of removing mastoid process for advanced parotid cancer on facial nerve identification, preservation and reconstruction

**DOI:** 10.1186/1746-160X-10-6

**Published:** 2014-03-03

**Authors:** Junkichi Yokoyama, Shinichi Ooba, Mitsuhisa Fujimaki, Takashi Anzai, Masataka Kojima, Katsuhisa Ikeda

**Affiliations:** 1 Department of Otolaryngology, Head and Neck Surgery, Juntendo University School of Medicine, 2-1-1,Hongo, Bunkyo-ku, Tokyo 113-8421, Japan

**Keywords:** Mastoid process resection, Temporal bone resection, Mastoidectomy, Advanced parotid cancer, Facial nerve preservation, Facial nerve reconstruction

## Abstract

**Background:**

Advanced parotid cancers more than 4 cm are firmly fixed around the main trunk of the facial nerve that can be hardly detected in narrow working space between mastoid process and parotid cancer. Even though facial nerve was preserved, facial nerve stretching during surgery has significantly serious effect on postoperative facial palsy.

**Objective:**

To evaluate usefulness of removing mastoid process in managing advanced parotid cancers to contribute identifying and preserving facial nerve.

**Method:**

The study was performed on 18 advanced parotid cancers which was more than 4 cm and invaded around the facial nerve. Thirteen cases were fresh cases and 5 were recurrent cases.

According to a modified Blair incision, the sternocleidomastoid muscle is detached from the mastoid process with electrocautery. When the mastoid process is removed, the main trunk of the facial nerve can be observed from stylomastoid foramen.

This procedure was evaluated based on the duration of surgery, working space, and postoperative facial nerve function.

**Results:**

In eleven cases, facial nerves were sacrificed. Negative margins were achieved in 100% of the patients. The mean duration for removing of the mastoid process to identify facial nerves was 4.6 minutes. The mean size of the removed mastoid process was 2.1 cm in height and 2.3 cm in width, and 1.8 cm in depth. The extended mean working space was 16.0 cm^3^, and, as a result, the tumors could be resected without retraction.

**Conclusion:**

Removing the mastoid process for advanced parotid tumors facilitates identification of the facial nerve and better preservation of the facial nerve function.

## Introduction

Advanced parotid cancers more than 4 cm are firmly fixed around the main trunk of the facial nerve that can be hardly detected in narrow working space between mastoid process and parotid gland with cancer. Though facial nerve is preserved, facial nerve stretching during surgery has significantly serious effect on postoperative facial palsy [1]. Parotid cancers can spread along the nerve to the temporal bone proximally. However, temporal bone surgeries for managing parotid malignancies are not well reported.

### Objective

To evaluate usefulness of removing mastoid process in managing advanced or recurrent parotid cancers to contribute identifying, preserving, and reconstructing facial nerve.

### Patients and method

The study was performed on 18 advanced parotid cancers of more than 4 cm and invading around the facial nerve. Thirteen cases were fresh cases and 5 were recurrent cases (Table 1).

**Table 1:** Patients and tumor characteristics

**Characteristics**	**Number**	**Characteristics**	**Number**
**Gender**		**Pathological type**	
Male	9	Carcinoma ex pleomorphic adenoma	4
Female	9	Salivary duct carcinoma	3
**Age**		Epithlial-myoepithlial carcinoma	2
Range	30-84	squamous cell carcinoma	2
Mean	59.3	Adenocarcinoma	2
Median	65	Acinic cell carcinoma	2
		others	3
**Previous treatment**			
Untreated	13	**HB scores**	
Recurrent	5	I	4
		II	5
**Stage**		III	2
Ill	3	IV	0
IVA	8	V	3
I’VE	7	VI	4

### Surgical procedure

**Figure 1:**
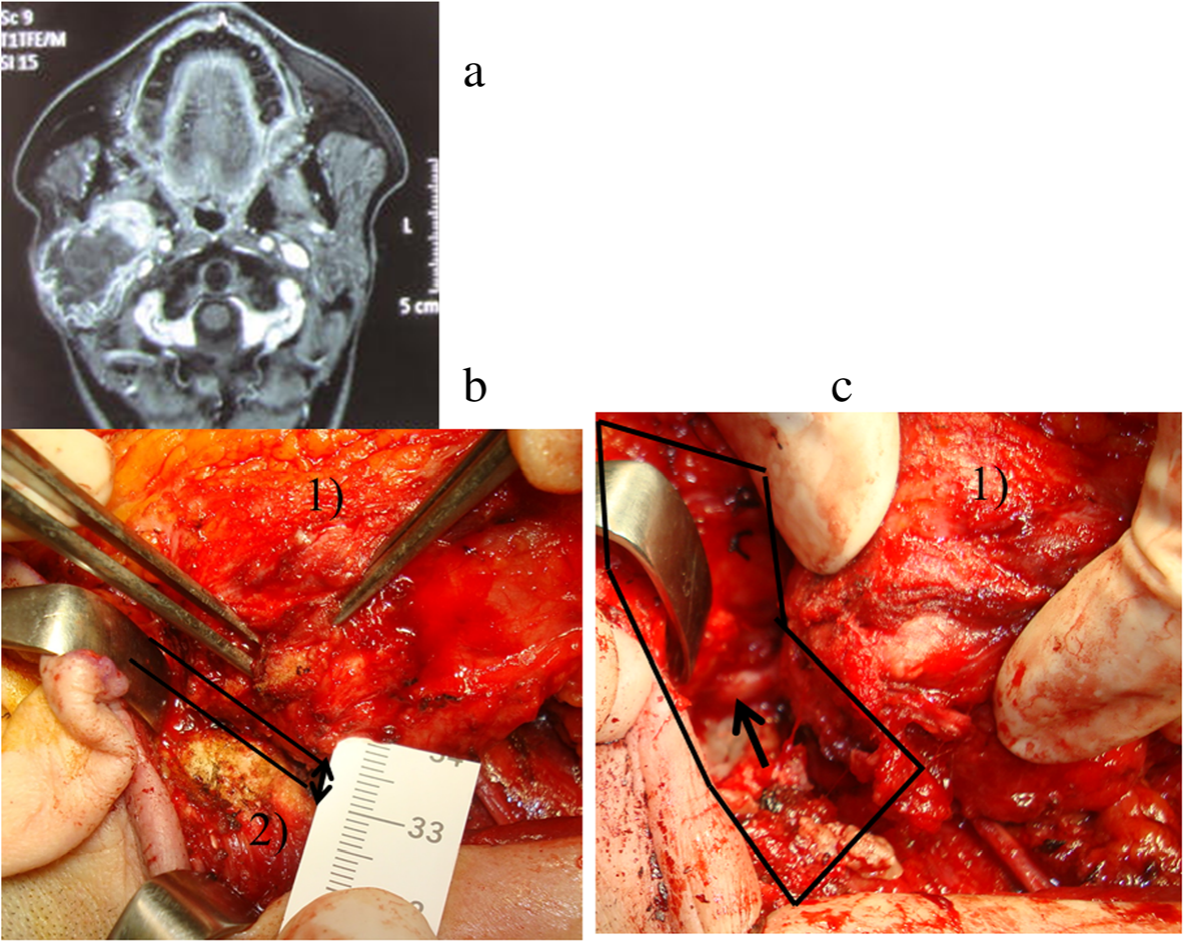
**Recurrent parotid cancer. a)**. MRI. **b)**. Intraoperative findings ( before removing mastoid process). Working space is significantly narrow (↔) between mastoid process and the tumor. 1) the tumor, 2) mastoid process. **c)**. After removing mastoid process. Expanding the working space facilitates identification of the facial nerve and dissection of parotid tumor without retracting the facial nerve (arrow).

According to a modified Blair incision, the skin flap is elevated in the superficial parotid fascia layer anteriorly to expose the mass to be resected. The parotid gland with cancer is separated from the cartilaginous external auditory canal. The sternocleidomastoid muscle is detached from the mastoid process with electrocautery. When the mastoid process is removed by large rongeur bone forceps, the main trunk of the facial nerve can be observed from stylomastoid foramen (Figure 1). The length of the cancer and mastoid process was 3 mm. The working space is very narrow. Removing mastoid process extends the working space extremely. This contributes to leveling the height between the facial nerve and the surface of the tumor, facilitating the handling of surgical instruments in the narrow working space along the facial nerve, and diminishing the tension on the facial nerve by releasing the facial canal proximally.

When the facial nerve has been already recognized as total paralysis, dissection is carried out along the main trunk of the facial nerve to the proximal intra-facial canal and distal to the cancer so that facial nerve reconstruction can be achieved after resection of the tumor. We accomplish this by removing the mastoid process and identifying the facial nerve in the vertical section of the temporal bone. Frozen examination of the proximal and distal nerve is checked prior to nerve reconstruction.

This procedure was evaluated based on the duration of surgery, working space, and postoperative facial nerve function using the House-Brackmann (HB) score [2].

## Results

Eighteen patients required either mastoidectomy (n: 15) or extended temporal bone resection (n: 3). In eleven cases, facial nerves were sacrificed. Theses facial nerves were reconstructed.

Negative margins were achieved in 100% of the patients.

**Figure 2:**
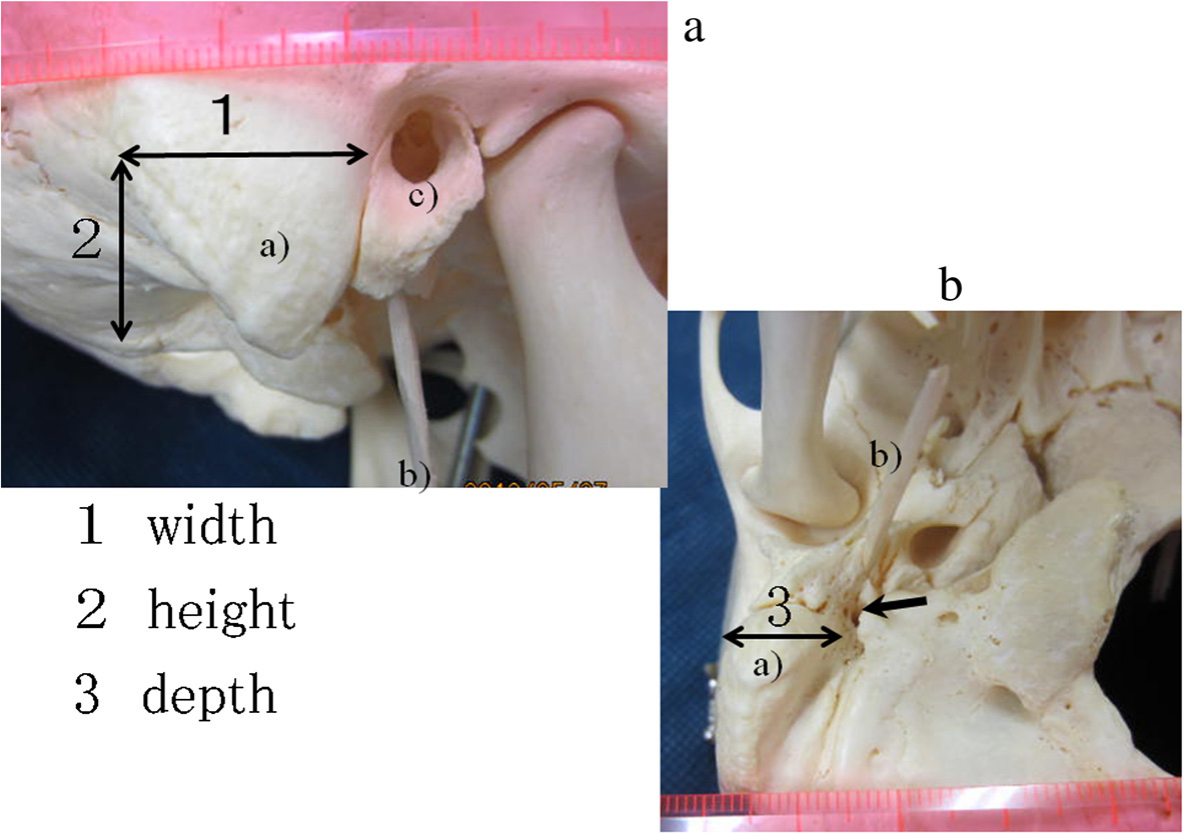
**Anatomy of mastoid process. a**. Lateral view. **b**. Inferior view a): mastoid process, b): styloid process, c): external ear.

The mean duration for removing of the mastoid process to identify facial nerves was 4.6 minutes (2 min-13 min). The mean length of the cancer and mastoid process was 4.3 mm. The mean size of the removed mastoid process was 2.1 cm in height and 2.3 cm in width, and 1.8 cm in depth (Figure 2). The extended mean working space was 16.0 cm^3^, and, as a result, the tumors could be resected without retraction (Table 2).

**Table 2:** Surgical procedure characteristics

**Characteristics**	**Number**	**Characteristics**	**Number**
**Tumor size**		**Removing size of mass**	cm
Range	4 cm — 8 cm	**Height**	
Median	5.2 cm	Range	1.9-2.4
		Mean	2.1
**Follow-up time**	months	**Width**	
Range	8-66	Range	2.1-2.8
Median	34.9	Mean	2.3
		**Depth**	
**Period of removing mastoid tip and identifying facial nerve**	minutes	Range	1.6-2.2
Range	2-13	Mean	1.8
Mean	4.6		
		**Working space**	cm3
**Distance between cancer and mastoid process**	mm	Range	13.5-19.8
Range	2--9	Mean	16
Mean	4.3		
		**postoperative HB scores**	
		I	4
		II	4
		III	3
		IV	5
		V	2
		VI	0

Preoperative each I, II, III, IV, V, and VI of H-B score was 4, 5, 2, 0, 3, 1 and 4, respectively.

Of the 7 patients in which the facial nerve was preserved, all 7 patients had almost normal facial function (HB I, II).

## Discussion

Facial nerve management is a crucial component of parotid surgery. Every effort should be made to preserve the facial nerve function [3]. Removal of the mastoid process and careful dissection around the nerve in the stylomastoid foramen permits full exposure of the nerve as it passes through the facial canal. As a result, of the 7 patients in which the facial nerve was preserved, all 7 patients had almost normal facial function (HB I, II). However, according to our historical cohort (without mastoid procedure), the overall incidence of facial paralysis (HB > 1) was 100% for temporary and 20% for permanent deficits.

We indicated this mastoidectomy for locally advanced parotid cancers larger than 4 cm that invaded firmly around the parotid, recurrent large parotid cancers, and complete facial paralysis in order to identify facial nerves without stretching and maintaining surgical margins.

Tumors involving only the mastoid process can be safely managed with removal of the mastoid process. Because large rongeur bone forceps are more useful and safer than electrical burr for head and neck surgeon lacking otology technique.

When cancers involve the middle ear, removing the mastoid process and resection the temporal bone along the facial nerve facilitates resection of the middle ear cancer. Inadequate surgical margins have been reported in up to 63% of patients [4]. Positive margins have been reported poor outcomes [3]. Our study showed 100% negative margins in the patients.

This procedure enabled safe identification of the facial nerve, and facilitated reconstruction in facial nerve in patients with sacrificed facial nerves.

## Conclusion

Removing the mastoid process for advanced parotid tumors facilitates identification of the facial nerve and, therefore, better preservation of the facial nerve function.

## Competing interests

The authors declare that they have no competing interests.

## Authors’ contributions

JY conceived of the study. JYprepared and edited the manuscript. SO and MF contributed to the acquisition of data. TA and MK performed the statistical analysis. JY and KI revised the final version of the manuscript. All authors read and approved the final manuscript
